# Blowhole tangential cecostomy and transanal tube insertion for neonatal cecal perforation in a patient with Hirschsprung’s disease in the earlier definitive operation era

**DOI:** 10.1186/s40792-019-0667-9

**Published:** 2019-07-10

**Authors:** Takeshi Shirai, Ryuichiro Hirose, Hiroki Kai, Kaori Inatomi, Yusuke Yanagi, Tsuyoshi Iwanaka, Akinori Iwasaki

**Affiliations:** 1 Department of General Thoracic, Breast and Pediatric Surgery, Faculty of Medicine, Fukuoka University, 7-45-1 Nanakuma, Jonan-ku, Fukuoka, Fukuoka 814-0180 Japan; 2 Department of Pediatric surgery, National Kyushu Medical Center, 1-8-1 Jigyohama, Chuo-ku, Fukuoka, Fukuoka 810-8563 Japan

**Keywords:** Tangential cecostomy, Neonatal intestinal perforation, Hirschsprung’s disease, Transanal indwelling tube

## Abstract

**Background:**

Although uncommon and seldom experienced, intestinal perforation is a well-known complication of Hirschsprung’s disease (HD). A literature review revealed that the cecum, including the appendiceal base, is a site of perforation. The cecum is not suitable for making an ordinary loop colostomy, and the optimal operative strategy remains to be established.

**Case presentation:**

We present a combination technique composed of tangential cecostomy at the perforated portion and postoperative care with a transanal indwelling tube, which was used in the treatment of a 3-day-old boy with cecal perforation with long-segment Hirschsprung’s disease. A temporary simple blowhole stoma and continuous decompression with daily irrigation via a transanal indwelling tube in the distal colon achieved a secure recovery and was followed by a definitive operation in the early period. The combination of tangential cecostomy and transanal indwelling catheter management led to the preservation of the ileocecal valve.

**Conclusions:**

We review the Japanese literature and emphasize the usefulness of this combination technique by blowhole tangential cecostomy and transanal tube insertion for neonatal cecal perforation in patients with HD in today’s early definitive operation era.

## Background

Hirschsprung’s disease (HD) is a common cause of distal bowel obstruction in the neonatal and infant period. Perforation of the intestine secondary to overdistention and/or enterocolitis is a well-known complication of HD. However, the actual occurrence of perforation in patients with HD is rare, and the incidence of intestinal perforation in HD was previously reported to be approximately 4% [1, 2].

A literature review revealed that the cecum, including the appendiceal base and appendix, is a frequent site of perforation. The cecum appears to be unsuitable for making an ordinary loop colostomy; thus, the optimal operative strategy remains to be established.

The authors herein report a case of cecal perforation in a 3-day-old male neonate with HD who was successfully treated with a temporary tangential cecostomy on the perforated site and management with an indwelling transanal tube, which enabled stable postoperative care followed by a definitive operation at 1 month of age.

This case is reported to highlight this surgical strategy of choice for cecal perforation in neonates with HD, which is especially useful in the earlier definitive operation era.

## Case presentation

**Fig. 1:**
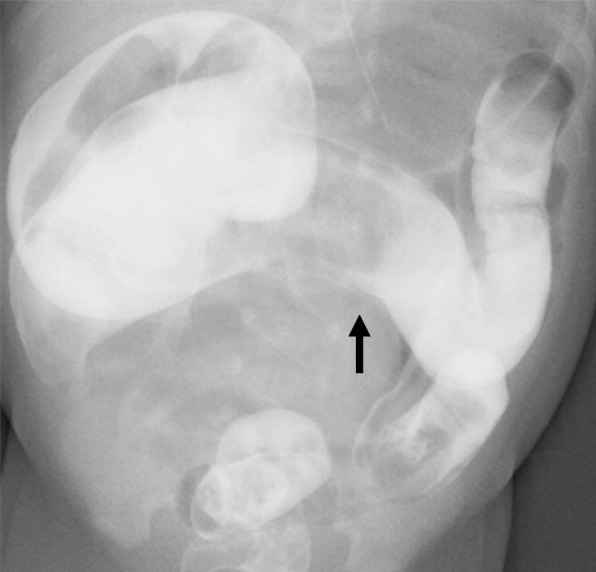
Gastrografin contrast enema: caliber change in the transverse colon (arrow)

A 2-day-old boy born at 40 weeks of gestation was admitted to our hospital due to repeated vomiting and abdominal distension. His birth weight was 2850 g. A blood test at admission revealed an elevation of the patient’s WBC count (26600/μL) and CRP (7.4 mg/dL) and lactate (18 mg/mL) levels. An X-ray photography showed dilatation from the ascending colon to the transverse colon. At this point, we doubted Hirschsprung’s disease-associated enterocolitis, and we started intravenous antibiotics therapy. Gastrografin contrast enema on the same day showed a caliber change in the transverse colon (Fig. 1). The 8.0 Fr ED tube was transanally indwelled into the hepatic flexure for continuous colonic decompression and repeated colonic irrigation was started. However, the following night, the patient’s condition showed an acute deterioration with marked abdominal distention and tachypnea. A blood test suggested inflammation with marked elevation of the patient’s CRP (17.9 mg/dL) and lactate (32 mg/dL) levels and acidemia (pH 7.20, BE − 5.7 mmol/L). We considered that his enterocolitis could not be controlled by antibiotics with decompression and irrigation via the transanal tube. The patient was brought to the operating theater for exploratory laparotomy.

**Fig. 2:**
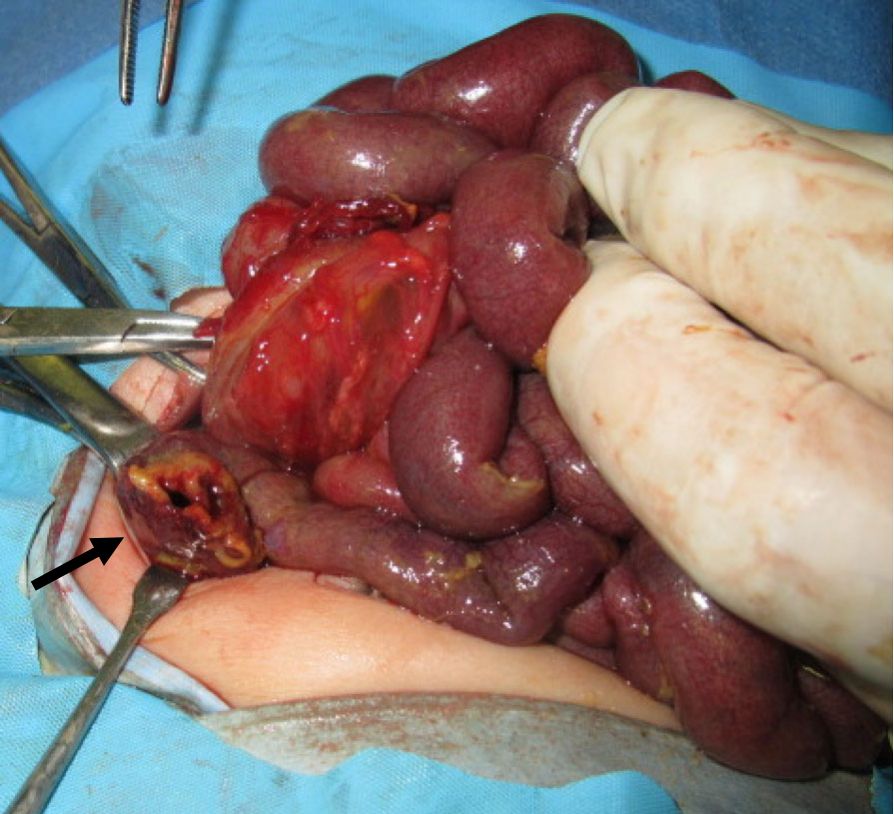
Laparotomy for cecal perforation (arrow) on the 3rd day of life

**Fig. 3:**
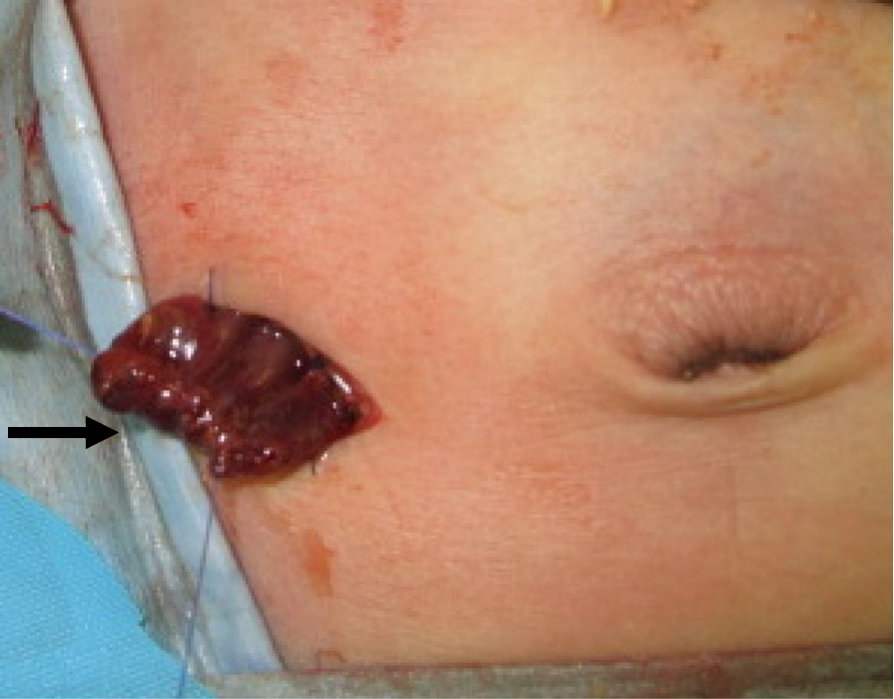
Blowhole tangential cecostomy by the perforation site of the cecum (arrow)

During laparotomy, perforation at the lateral cecal wall was detected with panperitoneal purulent ascites (Fig. 2). The tip of the indwelling tube was felt in the right transverse colon; thus, iatrogenic penetration by the catheter could be ruled out. A caliber change of the transition segment was observed at the mid-transverse colon. To minimize operative invasiveness as much as possible, we only performed leveling biopsies at the perforation site and the narrow segment of the left transverse colon. The perforated site of the cecal wall was exteriorized and sutured with minimal trimming as a tangential stoma (Fig. 3). Massive peritoneal lavage and silicon tube drainage were added. A few days later, a pathological examination revealed that the cecum specimen contained ganglion cells, while the left transverse colon did not.

**Fig. 4:**
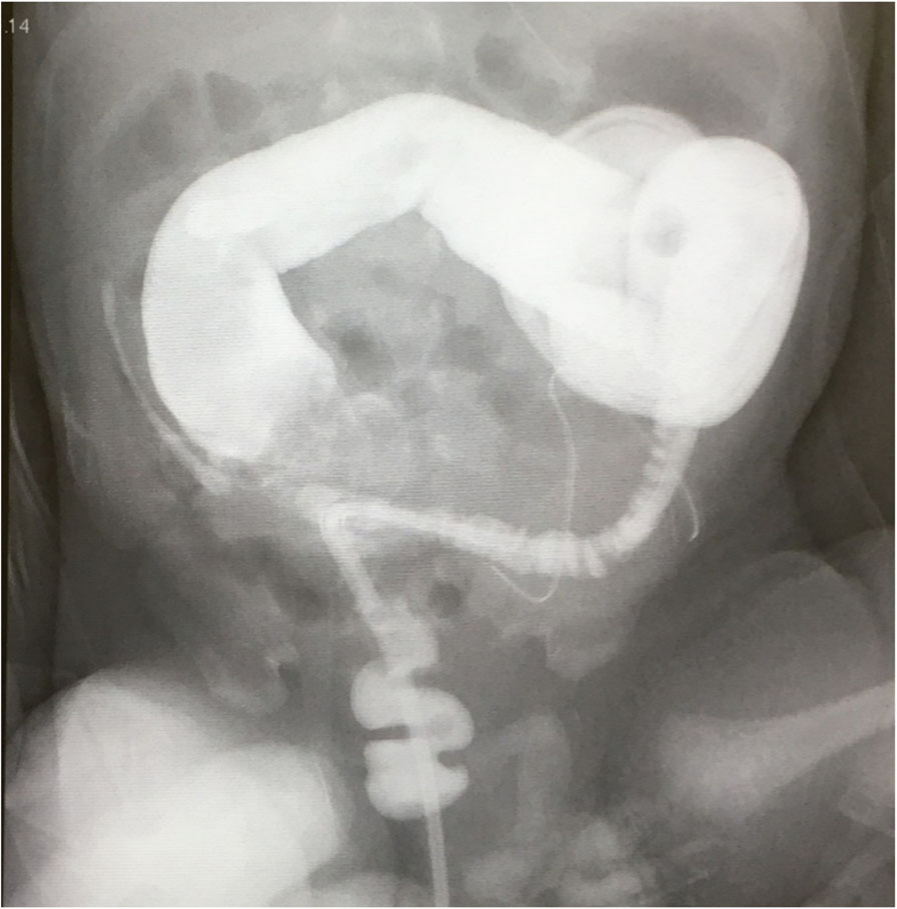
Management by transanal tube

The tangential cecostomy diverted the fecal stream constantly and the patient recovered well. The exteriorized cecal stoma shrank and transformed into a skin-level, cutaneous cecostomy, but good colonic decompression and diversion was maintained as a blowhole. On the 10th day after surgery, the transanal tube was re-indwelled for the secure management of aganglionic bowel (Fig. 4), and the patient continued usual oral feeding and showed body weight gain.

A definitive operation was performed at 47 days of age by the transverse incision of the first laparotomy with transanal approach. At the operation, there was the adhesion of peritonitis, but the cecostomy was simply closed by slight trimming without resection or anastomosis of the cecum. As a result, the ileocecal valve was preserved. The ganglionic ascending colon, confirmed by intraoperative histology, was pulled through and anastomosed to the anus by the Soave-Denda procedure. The postoperative course was unremarkable and the patient was discharged on the 60th day of life.

## Discussion

Intestinal perforation is a rare initial presentation in HD, which occurs in 3.2–4.4% of patients, mostly in the neonatal period [1, 2]. Newman et al. [2] reported that the most common sites of perforation were the cecum and ascending colon (68%), followed by the appendix (18%) and terminal ileum (6%). In cases with short- or long-segment aganglionosis, the perforation was proximal to or at the site of transition; however, in 84% of infants with total colonic aganglionosis (TCA), the perforation was located in the aganglionic bowel.

Twenty-nine cases of neonatal perforation in patients with HD were reported in the Japanese literature from 1998 to 2017.

After excluding cases of duodenal atresia, the mean gestational age, birth weight, and duration of perforation in 23 patients were 38.3 ± 1.6 SD weeks, 2999 ± 553 SD g, and 3.0 ± 1.8 SD days after birth. The most common sites of perforation were the cecum, ascending colon (*n* = 15), and ileum (*n* = 5). There were no patients with appendiceal perforation in this review. Various surgical procedures were selected, including ileostomy (*n* = 6), colostomy (*n* = 9), ileocecal resection and anastomosis (*n* = 3), and tangential cecostomy (our case; *n* = 1).

Newman et al. [2] warned that blind colostomy at the site of perforation might be an inappropriate treatment for cases in which the stoma site is located within the aganglionic bowel, as it might lead to inadequate decompression, continued obstruction, and a higher risk of enterocolitis. Thus, it is important that the underlying diagnosis of HD be suspected at the time of operation and that the infant treated accordingly.

In the present case, we performed cecostomy. Because the remarkable caliber change was recognized at the mid-transverse colon, we were sure that the real transition zone was located in the ascending or right transverse colon. In most reported cases of cecal perforation, colostomy at the perforated site or closure of the perforated site with ileostomy was selected. Staged definitive surgery has been performed with intraoperative leveling biopsy and stoma closure several months later. However, management and closure of the colostomy at the ascending colon or the ileocecal region seemed to be more difficult than tangential cecostomy. In some cases, a sacrifice of a segment of the ganglionic colon and/or ileocecal valve was required due to stenotic change near Bauhin’s valve and ileo-anal anastomosis was reluctantly performed at the time of the definitive operation.

Although the pathogenesis of intestinal perforation in HD is still unknown, most studies have proposed that inflammation may play a major role in its development at the site of bowel obstruction. In their review, Newman et al. reported that the mechanism of perforation appears to be directly related to increased luminal pressure from distal obstruction, as only a few cases showed signs of enterocolitis related to the perforation [2].

It was previously reported that the Law of Laplace dictates that the intraluminal pressure needed to stretch the wall of a hollow tube is inversely proportional to its radius; thus, the tension required to distend a hollow tube is lowest at the widest point. Clinically, this explains why the cecum is the most common site of perforation in cases of distal large bowel obstruction [3, 4].

During emergency laparotomy, the pediatric surgeon should be aware of this possibility and the cecum should be inspected for perforation at the time of surgery.

Cecostomy is a useful and less invasive surgical procedure for patients presenting with perforated cecum due to colonic obstruction; however, it is less frequently used and considered to be one of the oldest operations in surgery. In contrast to loop colostomy, a tangential-type stoma can act as a decompression and diversion window but cannot accomplish the complete division of the fecal stream. A transanal indwelling tube can manage the inflow feces and gas into the distal aganglionic bowel until definitive surgery [5–7]. Hirose et al. [6] reported that transanal catheter fixation enabled decompression and prevention of enterocolitis before surgery in 2 neonates with long-segment HD, which allowed the performance of the single-stage early transanal pull-through procedure with laparoscopy.

A recently published series suggests that the current era is characterized by earlier definitive surgery with increased utilization of laparoscopic and transanal approaches [8–11]. The combination of tangential cecostomy and transanal indwelling catheter management may be a useful strategy of choice which leads to preserve the ileocecal valve for the treatment of cecal perforation in patients with HD in the current early definitive operation era.

## Conclusions

The incidence of neonatal gastrointestinal perforation in patients with HD is thought to be decreasing. However, we hope that cecostomy and catheter management will be a helpful approach in the current early definitive operation era. The blowhole cecostomy procedure at the site of perforation may still be indicated for selected cases of colonic perforation in patients with HD.

## Data Availability

None.

## Abbreviations

HD: Hirschsprung’s disease
SD: Standard deviation
